# MicroRNA-34a modulates genes involved in cellular motility and oxidative phosphorylation in neural precursors derived from human umbilical cord mesenchymal stem cells

**DOI:** 10.1186/1755-8794-4-65

**Published:** 2011-09-19

**Authors:** Shing-Jyh Chang, Shun-Long Weng, Jui-Yu Hsieh, Tao-Yeuan Wang, Margaret Dah-Tsyr Chang, Hsei-Wei Wang

**Affiliations:** 1 Department of Obstetrics and Gynecology, Hsinchu Mackay Memorial Hospital, Hsinchu, 300, Taiwan; 2 Institute of Molecular and Cellular Biology, National Tsing Hua University, Hsinchu, 300, Taiwan; 3 Institute of Microbiology and Immunology, National Yang-Ming University, Taipei, 112, Taiwan; 4 Department of Pathology, Mackay Memorial Hospital, Taipei, 104, Taiwan; 5 Mackay Medicine, Nursing and Management College, Taipei, 112, Taiwan; 6 VGH-YM Genome Research Center, National Yang-Ming University, Taipei, 112, Taiwan; 7 Department of Education and Research, Taipei City Hospital, Taipei, 103, Taiwan

## Abstract

**Background:**

Mesenchymal stem cell (MSC) found in bone marrow (BM-MSCs) and the Wharton's jelly matrix of human umbilical cord (WJ-MSCs) are able to transdifferentiate into neuronal lineage cells both *in vitro *and *in vivo *and therefore hold the potential to treat neural disorders such as stroke or Parkinson's disease. In bone marrow MSCs, miR-130a and miR-206 have been show to regulate the synthesis of neurotransmitter substance P in human mesenchymal stem cell-derived neuronal cells. However, how neuronal differentiation is controlled in WJ-MSC remains unclear.

**Methods:**

WJ-MSCs were isolated from human umbilical cords. We subjected WJ-MSCs into neurogenesis by a published protocol, and the miRNome patterns of WJ-MSCs and their neuronal progenitors (day 9 after differentiation) were analyzed by the Agilent microRNA microarray.

**Results:**

Five miRNAs were enriched in WJ-MSCs, including miR-345, miR-106a, miR-17-5p, miR-20a and miR-20b. Another 11 miRNAs (miR-206, miR-34a, miR-374, miR-424, miR-100, miR-101, miR-323, miR-368, miR-137, miR-138 and miR-377) were abundantly expressed in transdifferentiated neuronal progenitors. Among these miRNAs, miR-34a and miR-206 were the only 2 miRNAs been linked to BM-MSC neurogenesis. Overexpressing miR-34a in cells suppressed the expression of 136 neuronal progenitor genes, which all possess putative miR-34a binding sites. Gene enrichment analysis according to the Gene Ontology database showed that those 136 genes were associated with cell motility, energy production (including those with oxidative phosphorylation, electron transport and ATP synthesis) and actin cytoskeleton organization, indicating that miR-34a plays a critical role in precursor cell migration. Knocking down endogenous miR-34a expression in WJ-MSCs resulted in the augment of WJ-MSC motility.

**Conclusions:**

Our data suggest a critical role of miRNAs in MSC neuronal differentiation, and miR-34a contributes in neuronal precursor motility, which may be crucial for stem cells to home to the target sites they should be.

## Background

Studies on implantation of human mesenchymal stem cells (MSCs) to treat neural disorders such as stroke or Parkinson's disease and stroke have shown promised potentials [1,2]. So far human bone marrow is the most common MSC source, but yield of MSC from bone marrow significantly decreases with donor age [3]. Many researchers thereby have searched for alternative sources of MSC in adult and extraembryonic tissues such as placenta, amniotic membrane, and umbilical cord. The Wharton's jelly part, a matrix of mucous connective tissue of umbilical cord surrounding and protecting umbilical cord arteries and vein, is enriched with fibroblast-like cells known as Wharton's jelly MSCs (WJ-MSCs) [4,5].

WJ-MSCs hold the potentials to transdifferentiate into neuronal lineage cells both *in vitro *and *in vivo *[2,4,6,7]. Cells cultured in low serum medium supplemented with basic fibroblast growth factor (bFGF) have been successfully induced to differentiate into glial cells and neurons *in vitro *[4]. A three-step method (neural induction, neural commitment and the neural differentiation step) could successfully induce *in vitro *neural differentiation of WJ-MSCs [2]. Fu and colleagues also successfully transdifferentiated WJ-MSCs into neurons *in vitro *by using neuronal conditioned medium (NCM) derived from the culture supernatants of 7-day postnatal Sprague-Dawley rat brains [6]. WJ-MSCs could further differentiate into dopaminergic neurons for treating Parkinsonian rats [8]. The *in vivo *neural differentiation ability of WJ-MSCs was demonstrated later by Weiss *et al*. that WJ-MSCs can transdifferentiate into cells of neuronal lineage *in vivo *by transplantation of WJ-MSCs in a hemiparkinsonian rat model [1]. *In vivo *neural differentiation of WJ-MSCs was further confirmed after intracerebral transplantation of WJ-MSCs in cerebral ischemic rats [2] or after transplantation into spinal cord transaction rats [9].

There have been studies focused on the neural differentiation mechanisms of MSCs. Wang and colleagues showed that the protein kinase A (PKA) signal transduction pathway mediates the neural differentiation of cord blood MSCs [10]. MicroRNAs (miRNAs) are a class of 18- to 24-nt, small, noncoding RNAs, which bind the 3' UTR of target mRNAs to mediate translational repression in cells [11-13]. MiRNAs have been shown to regulate cancer and developmental processes, such as stem cell self-renewal, neuronal differentiation, cell motility, and cell proliferation [14-20]. In bone marrow MSCs, miR-130a and miR-206 have been show to regulate the synthesis of neurotransmitter substance P in human mesenchymal stem cell-derived neuronal cells [21]. However, there is no study so far addressing the impacts of miRNAs on neural differentiation of WJ-MSC. Since MSCs from bone marrow and umbilical cord are quite distinct in terms of differentiation abilities and mRNA expression patterns [22], it is likely that miRNAs involved in WJ-MSC neural transdifferentiation are also distinct from those in BM-MSC neurogenesis.

Here, we report the first miRNA profile of undifferentiated human WJ-MSCs and WJ-MSC-derived neuronal precursors using a published differentiation protocol for BM-MSC [21]. MicroRNAs that were increased in the neuronal cells and decreased during neural differentiation were analyzed by bioinformatics algorithm to predict their mRNA targets and hence their function.

## Methods

### Isolation and cultivation of human MSCs

This research follows the tenets of the Declaration of Helsinki and informed consent was obtained from the donor patients. All human MSCs used for experiment were cultured for less than 8 passages in the MesenCult™ medium (StemCell Technologies, Vancouver, BC, Canada) in the presence of 5% CO_2_, or in Dulbecco's modified Eagle's medium (DMEM; Cat. 12100-061; Gibco-BRL, Paisley, U.K) supplemented with 10% fetal bovine serum (FBS; Cat. 12003; JRH Bioscience, KS, USA). MSCs from Wharton's jelly were collected as published [22]. Briefly, umbilical cords (UCs) were processed within 24 h after delivery. Wharton's jelly tissues were isolated from UCs and then digested with collagenase and trypsin. Cells were washed twice with PBS and plated on regular culture dishes in fresh culture medium, non-adherent cells were discarded on next day. Approximately 4 days to 1 week after expansion, fibroblast-like adherent cells were detached using trypsin-EDTA solution and then reseeded in fresh culture medium without dilution for further expansion (Passage 1: P1). Neural transdifferentiation was performed according to a published protocol [21]. Immunofluorescence assays (IFA) were carried as descried [23] using an anti-NeuN mAb (1:100; Millipore, Billerica, MA).

### MicroRNA microarray and data analysis

The Agilent Human miRNA Microarray Kit V2 (Agilent, Foster City, CA, USA) containing probes for 723 human microRNAs from the Sanger database v10.1 was used. GeneSpring GX 9 software (Agilent, USA) was used for value extraction. A 2-tailed Student's t-test was then used for the calculation of the *p *value for each miRNA probe. Heat maps were created by the dChip software http://www.dchip.org/. To predict the downstream mRNA targets of the miRNAs, the TargetScan web tool http://www.targetscan.org/ was used.

### Gene expression microarray probe preparation and data analysis

Total RNA collection, cRNA probe preparation, array hybridization and data analysis were done as previously described [24]. In brief, Affymetrix™ HG-U133 Plus 2.0 whole genome array was used. RMA log expression units were calculated from Affymetrix GeneChip array data using the 'affy' package of the Bioconductor http://www.bioconductor.org/ suite of software for the R statistical programming language http://www.r-project.org/. The default RMA settings were used to background correct, normalize and summarize all expression values. Significant difference between sample groups was identified using the method described by Storey & Tibshirani [24]. Briefly, a t-statistic was calculated as normal for each gene and a *p*-value then calculated using a modified permutation test [24]. To control the multiple testing errors, a false discovery rate (FDR) algorithm was then applied to these *p*-values to calculate a set of *q*-values: thresholds of the expected proportion of false positives, or false rejections of the null hypothesis. Gene annotation was performed by the ArrayFusion web tool http://microarray.ym.edu.tw/tools/arrayfusion/[25]. Gene enrichment analysis was performed by the Gene Ontology (GO) database using the WebGestalt http://bioinfo.vanderbilt.edu/webgestalt/ interface and the DAVID Bioinformatics Resources 6.7 database http://david.abcc.ncifcrf.gov/ by a hypergeometric test.

### RNA isolation and real-time quantitative polymerase chain reaction (qPCR)

Total RNA were extracted by Trizol (Invitrogen, Carlsbad, CA, USA) and 100 ng to 1 μg of total RNA were performed reverse transcription using the SuperScript™ III Reverse transcriptase kit (Invitrogen) as directed by the manufacturer. The expression of matured human miRNAs was determined by a stem-loop real-time PCR system^27^. The PCR reverse primer for miRNA was GTGCAGGGTCCGAGGT. The miRNA expression data were normalized to that of U6 snRNA, which was amplified with the specific forward and reverse primers 5'-CTCGCTTCGGCAGCAC-3' and 5'-AACGCTTCACGAATTTGCG-3', respectively. Real-time PCR reactions were performed using Maxima™ SYBR Green qPCR Master Mix (Cat. K0222; Fermentas, Glen Burnie, Maryland, USA), and specific products were detected and analyzed using a StepOne™ sequence detector (Applied Biosystems, USA).

### Transwell cell migration assay

Cell migration ability was evaluated using Costar Transwell^® ^Polycarbonate Permeable Supports (Corning, NY, USA). Briefly, 5 × 10^4 ^cells in 400 μl of culture medium were applied to the upper chamber of the device, and 600 μl of medium was added to the lower chamber. A polycarbonate membrane with a pore size of 8 μm was placed in between the two chambers. After 6 hours of incubation at 37°C, the membrane was fixed in 4% paraformaldehyde (Sigma-Aldrich) for 20 minutes at room temperature and then stained with Hoechst 33342 solution (Sigma-Aldrich) for 30 minutes. Migrated cells on the membrane were counted under a microscope.

## Results

### The miRNA signature associated with WJ-MSC neuronal transdifferentiation

**Figure 1:**
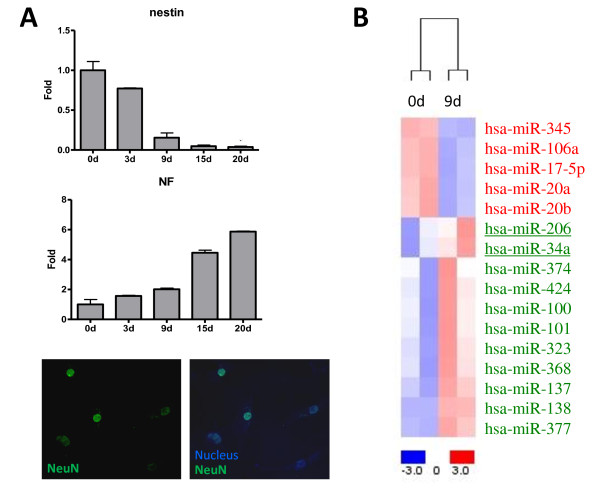
**MiRNome analysis of umbilical cord MSCs and differentiated neural precursors**. (**A**) (*upper and middle*) Histograms showing the induction of neural differentiation in WJ-MSCs. WJ-MSCs were subjected into neurogenesis, and total RNAs were collected at the indicated time points for real-time PCR validation. neurofilament (NF), neurogenesis-related genes; nestin, stemness gene. (*lower*) Immunofluorescence assays on NeuN protein. Day 3 neuronal precursors derived from WJ-MSC were used. (**B**) A heat map shows the miRNA expression patterns in WJ-MSCs (0d) and derived neural precursors which were differentiated for 9 days (9d). MiRNAs in red, increased expression; in blue, decreased.

WJ-MSCs were subjected into neurogenesis as shown before [21]. Total RNAs were collected at day 0, 3, 9 15 and 20, and real-time PCR validation of a neurogenesis-related gene, neurofilament (NF), and a stemness marker, nestin, were performed (Figure 1A). As expected, WJ-MSCs gradually lost their stemness while gained neuronal features (Figure 1A, upper and middle panels). NeuN, a neural specific marker in vertebrate [26], could be detected in differentiated cells 3 days after induction (Figure 1A, bottom panel).

**Figure 2:**
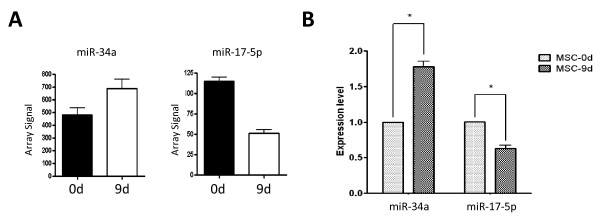
**Validation of miRNA array data**. (**A**) Array hybridization signals of miR-34a and miR-17-5p. (**B**) Mean expression levels of miR-34a and miR-17-5p were compared to that of U6 small nuclear RNA control. Results were done by real time RT-PCR and expressed as the mean ± standard deviation.

We then analyzed the global miRNA expression patterns (the "miRNome") of WJ-MSCs and their neuronal progenitors by microarray analysis. Since we aim to identify miRNAs associated with the early events of neuronal transdifferentiation, total RNAs were collected at day 0 and day 9 post differentiations. Differentially expressed miRNAs were identified by 2-tailed Student's t-test with a significance level of P < 0.05 plus ≧ 1.5-fold change. A heat map for these miRNAs indicates their unique expression levels between each cell group (Figure 1B). Five miRNAs were enriched in WJ-MSCs, including hsa-miR-345, hsa-miR-106a, hsa-miR-17-5p, hsa-miR-20a and hsa-miR-20b (Figure 1B). In contrast, 11 miRNAs (hsa-miR-206, hsa-miR-34a, hsa-miR-374, hsa-miR-424, hsa-miR-100, hsa-miR-101, hsa-miR-323, hsa-miR-368, hsa-miR-137, hsa-miR-138 and hsa-miR-377) were abundantly expressed in day 9 neuronal progenitors (Figures 1B and 2A).

Among miRNAs up-regulated during WJ-MSC neuronal differentiation, miR-34a and miR-206 were also up-regulated in neuronal precursors derived from BM-MSC [21] (Figures 1B, underlined). It has been confirmed previously that miR-206 can regulate the synthesis of neurotransmitter substance P in human BM-MSC-derived neuronal cells [21]. However, roles of miR-34a in neuronal precursor cells are as yet unclear. We confirmed the up-regulation of miR-34a in day 9 neuronal progenitors by qPCR (Figures 2B). The reduction of miR-17-5p during neurogenesis was also confirmed (Figures 2B).

### Functional module analysis as a framework for the interpretation of miR-34a biology

**Figure 3:**
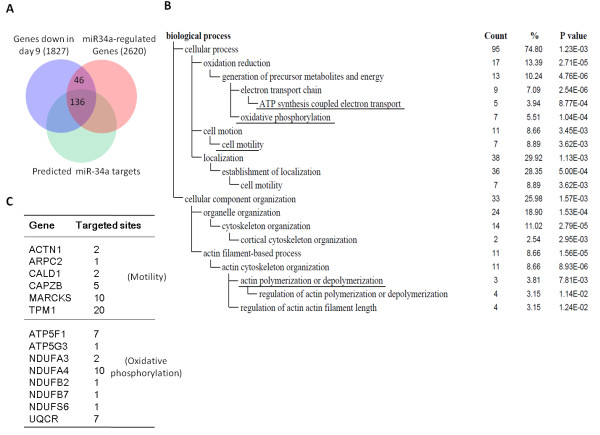
**Gene expression microarray analysis for finding miR-34a-regulated genes and biological modules**. (**A**) A Venn diagram shows that 136 miR-34a-affected genes are decreased in 9d precursors [27]. These genes all contain putative miR-34a binding sites revealed by the miRTar webtool http://mirtar.mbc.nctu.edu.tw/human/. (**B**) The above 136 genes were subjected to the GO database search via the DAVID Bioinformatics Resources 6.7 database http://david.abcc.ncifcrf.gov/. These categories were selected from the Biological Process organizing principle in the GO project http://www.geneontology.org/. The number of genes and P values for each category are also listed. Underlined: discussed in the text. (**C**) Motility and oxidative phosphorylation genes (mapped by the WebGestalt http://bioinfo.vanderbilt.edu/webgestalt/ interface) which are targeted by miR-34a. Numbers of targets sites within the 3' UTR of each gene are shown.

To increase our knowledge on miR-34a biology and to better explain miR-34a function, we analyzed by microarray the transcriptome of cells transfecting with miR-34a oligos for finding miR-34a-regulated mRNAs. As a result, we identified 136 miR-34a-suppressed genes were also down-regulated in day 9 neuronal progenitors (Figure 3A; day 9 neurogenesis genes were from a published reference [27]). These 136 genes all possessed putative miR-34a-binding sites (Figure 3A). To understand more how these 136 genes might be correlated with neuronal progenitor biology and to provide quantitative evidences, these 136 genes were subjected into the Gene Ontology (GO) database search [28] to find statistically over-represented functional groups within them. Given that the whole human transcriptome was represented in microarray performed, this analysis was not biased toward the coverage of the microarray.

The GO categories of biological processes being statistically overrepresented (P < 0.05) among the 136 newly identified miR-34a target genes are shown in Figure 3B. Categories associated with cell motility, energy production (including those with oxidative phosphorylation, electron transport and ATP synthesis), and actin cytoskeleton organization were all significantly (P < 0.05) repressed by miR-34a (Figure 3B, underlined). Details of miR-34a-targeted genes involved in cell motility or oxidative phosphorylation are listed in Figure 3C.

### Suppression of MSC motility by miR-34a

**Figure 4:**
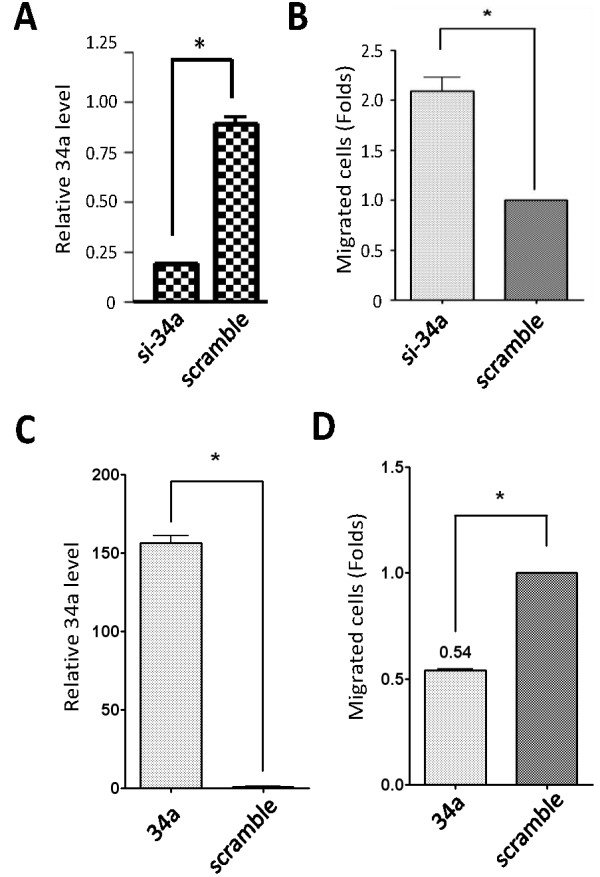
**MiR-34a regulates cell motility**. (**A-B**) WJ-MSCs were transfected with siRNA against miR-34a, or with a scramble siRNA negative control. MiR-34a expression levels were detected by real time RT-PCR (**A**), and the relative cell migration ability was measured at 48 h posttransfection (**B**). Data shown represent an average of 10 wells. Asterisks, P < 0.05. (**C-D**) WJ-MSCs were transfected with miR-34a or with scramble oligonucleotides. MiR-34a levels were detected by real time PCR (**C**) and the relative cell migration ability was measured at 48 h posttransfection (**D**).

MiR-34a has been linked to reduced cell motility in cancer cells [15]. The suppression of cytoskeleton-related genes and cell motility genes indicates that miR-34a may also regulate MSC motility. The suppression of ATP-synthesis genes by miR-34a further indicates this possibility. We examined our hypothesis by manipulating miR-34a levels in MSCs. We knocked down endogenous miR-34a expression in MSCs and performed cell motility assays. As shown in Figure 4, WJ-MSC motility was increased (Figure 4B) when the endogenous miR-34a level was reduced (Figure 4A). On the contrary, cellular motility was reduced when miR-34a was overexpressed in cells (Figures 4C-D). Of note, cell viability was not significantly affected by knocking down or overexpressing miR-34a in MSCs (not shown).

## Discussion

MSCs isolated from various anatomic locations exhibit immune-suppressive properties, follow a pattern of multilineage differentiation, including neuronal transdifferentiation [29,30]. MSCs thereby provide an alternate source of neuronal cells for tissue repair, with potential for autologous transplantation. MicroRNAs (miRNAs) are small RNAs of 18-24 nucleotides in length that are involved in the regulation of gene expression and hence a variety of biological processes, including stem cell differentiation, through post-transcriptional RNA interference-based mechanisms [11-13,21]. How miRNAs are involved in MSC neurogenesis or in the activities of differentiated neuronal precursors remain largely unclear.

It has been reported that in BM-MSC-derived neurons (day 12), 32 miRNAs are altered, including miR-206 and miR-34a [21]. In this differentiation system, MSC-derived neurons express the neurotransmitter gene, Tac1, at the level of transcription, but without translation to its encoded neurotransmitter substance P (SP) [21]. However, stimulation with the proinflammatory mediator, IL-1α, resulted in Tac1 mRNA translation. IL-1α thereby alleviates translational repression of Tac1 mRNA through negative effects on miRNAs. MiR-206 is one of the miRNAs down-regulated by IL-1α, while miR-34a is still abundant in day 12 neurons in the presence or absence of IL-1α [21]. This data indicates that miR-34a is involved in functions other than SP production. In this work we show that miR-34a modulates genes involved in cellular motility and oxidative phosphorylation in WJ-MSC-derived neural precursors. These clues came from microarray analysis on cells overexpressing miR-34a (Figure 3). Of note, microarray experiment will only identify genes that are regulated at the mRNA stability levels by miR-34a and not the genes that are regulated through changes in translation. The direct inhibition of miR-34a downstream targets by miR-34a, however, is still to be test by more direct assays such as 3'-UTR reporter assays and immunoblotting. We proved that miR-34a contributes in cell motility in WJ-MSCs. Whether miR-34a also regulates BM-MSC motility and ATP production is under investigation.

Cell motility plays a crucial role in stem cell-based therapy. In a middle cerebral artery occlusion (MCAO) rat model, engrafted WJ-MSCs could migrate to the penumbric area to repair the ischemic boundary zone and differentiated into glial, neuronal, doublecortin+, CXCR4+, and vascular endothelial cells to enhance neuroplasticity in the ischemic brain [2]. Knocking down endogenous miR-34a in transplanted MSCs may enhance cell motility and energy production, thereby favoring engrafted WJ-MSCs or their neuronal progenitors to locate to injured locations *in vivo*.

The expression of miR-34a is regulated by p53, and introducing miR-34a into cancer cells results in apoptosis, senescence and/or cell-cycle arrest [31]. In prostate cancer, miR-34a inhibits cancer stem cells and metastasis by directly repressing CD44 [32]. miR-34a is also tumor suppressive in brain tumors and glioma stem cells and can induce cancer cell differentiation [16]. However, miR-34a is overexpressed in various types of human cancers and can support cell proliferation in renal carcinogenesis [14]. The roles of miR-34a in different cell types are therefore different, which may due to the differential expression of miR-34a target mRNAs in a different context. In mouse embryonic stem cells (ESCs), the suppression of miR-34a by anti-miR caused the block of ESC differentiation induced by LIF withdrawal [33]. For the bipotent K562 human leukemia cells, miR-34a is strongly up-regulated during phorbol ester-induced megakaryocyte differentiation [34]. Whether niR-34a is also involved in BM-MSC and WJ-MSC differentiation is another interesting direction to follow.

In summary we have identified miRNome changes during WJ-MSC transdifferentiation into neurons and have found novel miR-34a downstream targets. The direct inhibition of those miR-34a downstream targets by miR-34a, however, is still to be test by more direct assays such as 3'-UTR reporter assays and immunoblotting. Roles of other filtrated miRNAs in MSC neuronal differentiation are also waited to be checked in the future. We also found that 4 miRNAs belonging to the miR-17 family (that is, miR-20a, miR-20b, miR-17-5p and miR-106a) are down-regulated during WJ-MSC neurogenesis (Figures 1, 2). How these miRNAs work together in maintaining stemness will be a critical issue to address.

## Conclusions

We have identified miRNome changes during WJ-MSC transdifferentiation into neurons and have identified the miRNome pattern during the neurogenesis of WJ-MSC. MiR-34a and miR-206 were the only 2 miRNAs which were commonly down-regulated during neuronal differentiation in both WJ-MSC and BM-MSC. MiR-34a suppressed the expression of 136 neuronal progenitor genes, and gene enrichment analysis showed that these genes were associated with cell motility and energy production. *In silico *data were verified by wetlab experiments, which proved that miR-34a can inhibit WJ-MSC motility. Our data suggest a critical role of miRNAs in MSC activities and neuronal differentiation, and miR-34a contributes in neuronal precursor motility, which may be crucial for stem cells to home to the target sites they should be.

## Competing interests

The authors declare that they have no competing interests.

## Authors' contributions

SJC, SLW, MDC, and HWW contributed study concepts. SJC, SLW, and HWW designed the study project. SJC and SLW collected microarray data sets. SJC and JYH executed project plan and data analysis. SJC, SLW, TYW, and HWW carried out data interpretation and discussion. SJC wrote the manuscript. SLW, JYH, TYW, and MDC revised it. Then HWW finalized this manuscript. HWW administrated and financial supported this study. All authors read and approved the final manuscript.

## Pre-publication history

The pre-publication history for this paper can be accessed here:

http://www.biomedcentral.com/1755-8794/4/65/prepub
